# Involvement of microRNAs in physiological and pathological processes in the lung

**DOI:** 10.1186/1465-9921-11-159

**Published:** 2010-11-23

**Authors:** Tereza Tomankova, Martin Petrek, Eva Kriegova

**Affiliations:** 1 Laboratory of Immunogenomics and Proteomics, Institute of Molecular and Translational Medicine, Medical Faculty Palacky University Olomouc, the Czech Republic

## Abstract

To date, at least 900 different microRNA (miRNA) genes have been discovered in the human genome. These short, single-stranded RNA molecules originate from larger precursor molecules that fold to produce hairpin structures, which are subsequently processed by ribonucleases Drosha/Pasha and Dicer to form mature miRNAs. MiRNAs play role in the posttranscriptional regulation of about one third of human genes, mainly via degradation of target mRNAs. Whereas the target mRNAs are often involved in the regulation of diverse physiological processes ranging from developmental timing to apoptosis, miRNAs have a strong potential to regulate fundamental biological processes also in the lung compartment. However, the knowledge of the role of miRNAs in physiological and pathological conditions in the lung is still limited. This review, therefore, summarizes current knowledge of the mechanism, function of miRNAs and their contribution to lung development and homeostasis. Besides the involvement of miRNAs in pulmonary physiological conditions, there is evidence that abnormal miRNA expression may lead to pathological processes and development of various pulmonary diseases. Next, the review describes current state-of-art on the miRNA expression profiles in smoking-related diseases including lung cancerogenesis, in immune system mediated pulmonary diseases and fibrotic processes in the lung. From the current research it is evident that miRNAs may play role in the posttranscriptional regulation of key genes in human pulmonary diseases. Further studies are, therefore, necessary to explore miRNA expression profiles and their association with target mRNAs in human pulmonary diseases.

## A. miRNA definition, biology and function

### Discovery of microRNA (miRNA)

*lin-4 *was the first short non-coding RNA discovered in 1993 as a regulator of developmental timing in *Caenorhabditis elegans *[1]. The first non-coding RNA identified in humans was *let-7*, which has been found involved in the control of developmental timing in humans and animals [2,3]. Soon it became evident that these short non-coding RNAs are a part of much larger class of non-coding RNAs and the term microRNA (miRNA) was introduced [4]. To date, more than 900 miRNAs in *Homo sapiens *have been identified (940 in miRBase v15).

### Structure and function of miRNAs

MiRNAs are small non-coding RNAs ~22 nucleotides (nt) long involved in the negative post-transcriptional gene regulation via RNA interference mechanism [5,6]. The sequences of miRNAs are highly conserved among plants-microorganisms-animals, suggesting that miRNAs represent a relatively old and important regulatory pathway [7]. MiRNAs belong to the most abundant class of human gene regulators [8]: up to a third of the human genes are regulated by miRNAs [9]. MiRNAs are, therefore, key regulators of numerous genes in biological processes ranging from developmental timing to apoptosis [e.g. [10-14]]. It has been speculated that miRNAs may be associated with the regulation of almost every aspect of cell physiology [8].

### miRNA biogenesis

**Figure 1:**
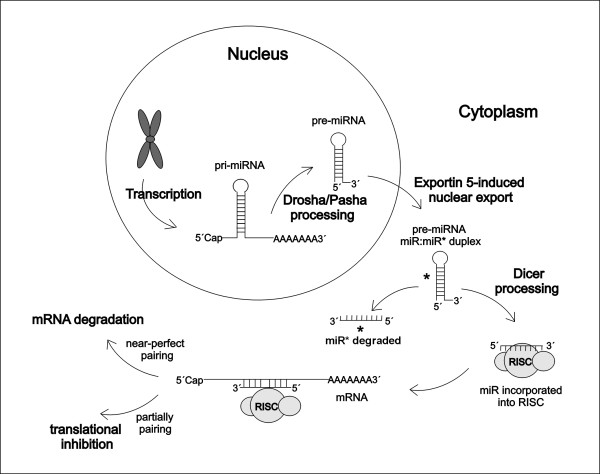
**miRNA biogenesis**. MiRNAs are transcribed by RNA polymerase II from the genomic DNA as long (hundred or thousand nucleotides) primary miRNA transcripts (pri-miRNAs). A local stem-loop structure of pri-miRNAs is then cleaved in the nucleus by the dsRNA-specific ribonuclease Drosha/Pasha to produce a 70 nucleotides long precursor miRNA (pre-miRNA). Pre-miRNAs in form of hairpins are then actively transported from the nucleus to the cytoplasm. In the cytoplasm, pre-miRNAs are subsequently cleaved by RNase III Dicer into ~22-nt miRNA duplexes, consisting of the "guide" (miR) strand and the "passenger" (miR*) strand. The "passenger" strand is degraded, the "guide" strand is incorporated into the RNA-induced silencing complex (RISC) and serves as a functional, mature miRNA, acting by two different mechanisms according to the complementarity with the target mRNA. Adopted from Kim [15].

MiRNA genes are localized in the non-coding regions or in the introns of protein-coding genes in the genomic DNA. The miRNA genes are much longer than biologically active, mature miRNAs which originate through a multi-step process [15] (Figure 1). Briefly, transcription by the RNA polymerase II leads to hundred or thousand nucleotides long primary miRNA transcripts (pri-miRNAs) [16]. A local stem-loop structure of pri-miRNAs is then cleaved in the nucleus by the dsRNA-specific ribonuclease Drosha/Pasha to 70 nucleotides long precursor miRNA (pre-miRNA) [17] in a process known as "cropping" [18,19]. Pre-miRNAs are then actively transported from the nucleus to the cytoplasm [20,21]. In the cytoplasm, pre-miRNAs are subsequently cleaved by RNase III Dicer into ~22-nt miRNA duplexes [17,20]. One strand of the short-lived miRNA duplex is degraded ("passenger" strand, miR*), whereas the other ("guide", miR) strand is incorporated into the RNA-induced silencing complex (RISC) and serves as a functional, mature miRNA [8]. Selection of the "guide" strand is based on the base pairing stability of both dsRNA ends [22,23].

### Mechanism of miRNA action

**Figure 2:**
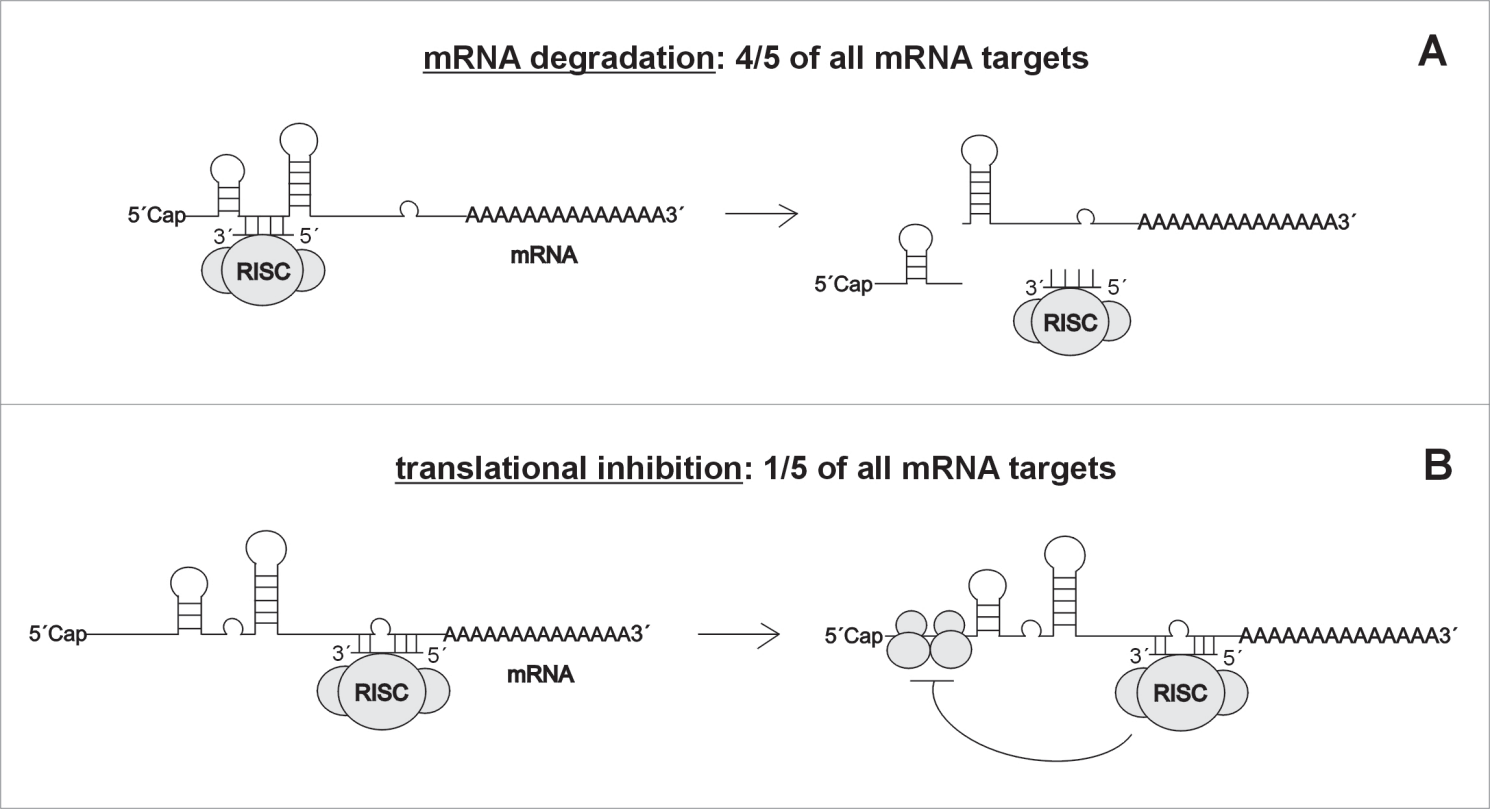
**Mechanism of miRNA action on target mRNA**. According to the complementarity between miRNA and 3' untranslated region (UTR) of target mRNA, there are two mechanisms of miRNA action: (**A**) when miRNA is near-perfectly complementary with target mRNA, deadenylation and subsequent degradation of the target mRNA occurs (major mechanism of miRNA action); (**B**) when miRNA is only partially complementary to its target mRNA, translational inhibition occurs. Adopted from Brodersen et al [24].

Depending on the complementarity between miRNA and 3' untranslated region (UTR) of target mRNA there are two known mechanisms of miRNAs action on mRNAs: 1) target mRNA degradation and 2) translational inhibition with little or no influence on mRNA levels [24] (Figure 2). Firstly, the deadenylation and subsequent degradation of the target mRNA occurs when miRNA is near-perfectly complementary with target mRNA [25,26]. A recent study proved that mRNA degradation represents the major mechanism of miRNA regulation [27]. The authors showed that about 84% of all protein-coding mRNA targets undergo degradation while recognized by their cognate miRNA [27]. Secondly, the translational inhibition occurs when miRNA is only partially complementary to its target mRNA [28-30]. In light of the recent study by Guo et al [27], this mechanism does not represent a predominant reason for reduced protein output.

Besides the complementarity between miRNA and mRNA, several other factors may influence the miRNA action such as impaired processing, methylation, gene polymorphisms, gene amplification, deletion of Dicer, translocations and others [31].

### Targets of miRNAs

It is evident that single miRNAs may regulate translation of numerous downstream mRNAs and each mRNA is likely to be regulated by several miRNAs simultaneously [30,32]. Thus, identification of miRNA target genes has been a great challenge [33]. Numerous computational algorithms [34-43] were established which combined 5' seed matches, thermodynamic stability and conservation analysis in order to maximize specificity when predicting mRNA targets [44] (Table 1). Nevertheless, various algorithms differ in the selection of mRNA targets and simultaneous application of several algorithms is, therefore, highly recommended. Nowadays, many web-based applications [45-52] have been developed by combining existing prediction programs with functional annotations associated to many miRNA, gene, protein or biological pathway resources such as miRBase, Ensembl, Swiss-Prot, UCSC genome browser, KEGG pathway and other databases [44] (Table 2).

**Table 1:** Computational algorithms for miRNA target prediction.

Algorithm	Web link	References
miRanda	http://www.microrna.org	[34]

TargetScan	http://genes.mit.edu/targetscan	[35]

TargetScanS	http://genes.mit.edu/targetscan	[36]

PicTar	http://pictar.mdc-berlin.de	[37]

DIANA-microT	http://diana.pcbi.upenn.edu/cgi-bin/micro_t.cgi	[38]

ElMMo	http://www.mirz.unibas.ch/ElMMo2/	[39]

MirTarget2	http://mirdb.org	[40]

miTarget	http://cbit.snu.ac.kr/~miTarget/	[41]

rna22	http://cbcsrv.watson.ibm.com/rna22.html	[42]

RNAhybrid	http://bibiserv.techfak.uni-bielefeld.de/rnahybrid	[43]

Adopted from Min et al [44].

**Table 2:** Methods with extended features for miRNA target prediction.

Name	Web link	References
GOmir	http://www.bioacademy.gr/bioinformatics/projects/GOmir	[45]

miRDB	http://mirdb.org	[46]

miRecords	http://miRecords.umn.edu/miRecords	[47]

miRGator	http://genome.ewha.ac.kr/miRGator	[48]

miRNAMap	http://miRNAMap.mbc.nctu.edu.tw	[49]

mirZ	http://www.mirz.unibas.ch	[50]

MMIA	http://156.56.93.156/~MMIA/mmia_main.html	[51]

TarBase5.0	http://diana.cslab.ece.ntua.gr/tarbase	[52]

Adopted from Min et al [44].

However, because of high similarities in miRNA sequences, computational algorithms may predict a large number of putative miRNA binding sites on mRNA targets [33]. Thus, experimental validation in biological system is fundamental to complete the target prediction study [44]; the currently available methods [53-67] are listed in Table 3. Of these, antagomir studies or immunoprecipitation of Ago-bound mRNAs have been specifically developed for miRNA-mRNA studies. Antagomirs represent a novel class of chemically engineered oligonucleotides used to silence endogenous microRNAs [64,65]. Immunoprecipitation is then based on the observation that each member of the Argonaute (Ago) protein family (catalytic components of the RNA-induced silencing complex) can bind to miRNAs and to partially complementary sequences in the 3'-UTR of specific target mRNAs. Thus, using highly specific monoclonal antibodies against members of the Ago protein family, Ago-bound mRNAs can be co-immunoprecipitated [66,67].

**Table 3:** Experimental methods to check the functional interaction between miRNA and target mRNA.

Method	Selected references
Luciferase reporter assay	[53]

Northern blot analysis	[54]

Quantitative real-time PCR	[55]

Ribonuclease protection assay	[56]

*in situ *hybridization	[57,58]

miRNA mimics	[59]

Western blot	[60]

Immunocytochemistry	[61]

Bead-based flow cytometry method	[62]

Suppression of miRNA expression in cells by anti-sense locked-nucleic acid oligonucleotides	[63]

Antagomir assays	[64,65]

Immunoprecipitation of Ago-bound mRNAs	[66,67]

## B. miRNA in pulmonary physiology and pathology

### Role of miRNAs in the lung

**Figure 3:**
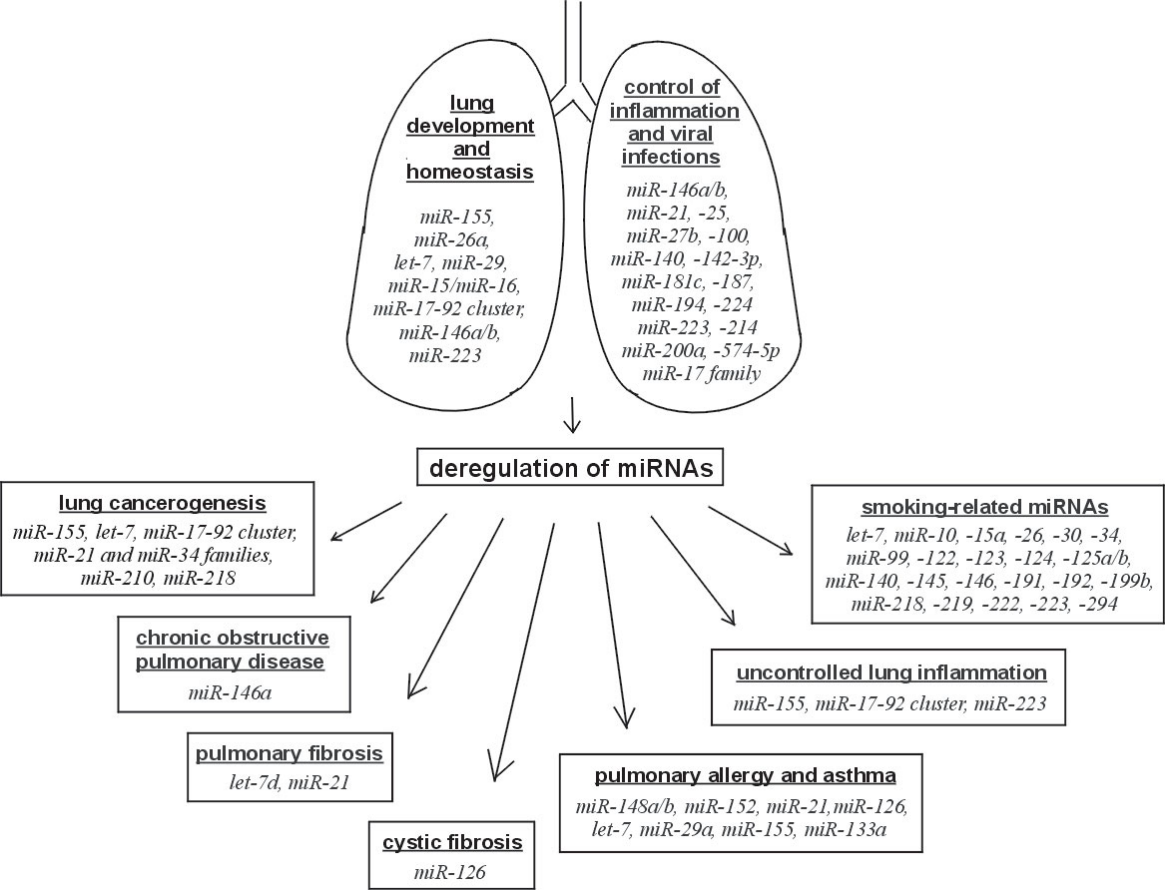
**Involvement of miRNAs in physiological and pathological processes in lung**. The scheme shows several miRNAs involved in physiological and pathological processes in lung. There has been shown that numerous miRNAs are implicated in maintaining lung homeostasis and development. When deregulation of these miRNAs occurs, pathological processes turn up and may lead to the development of pulmonary diseases.

The lung has a very specific miRNA expression profile, highly conserved across mammalian species [68,69]. However, the knowledge of the role of miRNAs in physiological and pathological conditions in the lung compartment is still limited and it is based mainly on the studies in animal models. MiRNAs have been shown to be involved in 1) the lung development and homeostasis, 2) in inflammation and viral infections and 3) miRNA deregulation may contribute to several pulmonary diseases (Figure 3). Hereby, we summarize the knowledge of the involvement of miRNAs in the lung and current information on their posttranscriptional regulation ongoing in the lung compartment. Besides pathology we pay attention also to physiological lung because understanding miRNA function in normal condition is prerequisite to description of its involvement in disease.

### MiRNAs in the lung in physiological condition

#### Homeostasis and lung development

Several miRNAs such as *miR-155, miR-26a*, *let-7, miR-29, miR-15/miR-16, miR-223, miR-146a/b *and the *miR-17-92 cluster *have been shown to be involved in homeostasis and in the lung development (Table 4). The pulmonary role of *miR-155 *was studied in murine lung, where it has been shown that *miR-155 *is crucially involved in the differentiation of naive T-cells into Th1 and Th2 cells [70,71]. Mice deficient in *bic/miR-155 *became immunodeficient and displayed increased lung remodelling, higher bronchoalveolar leukocytes and impaired T- and B-cell responses to inflammatory stimuli [70]. Another member of miRNA family, *miR-26a*, has been shown to be selectively expressed in the bronchial and alveolar epithelial cells in murine lung [72]. Target mRNA of *miR-26a *is the transcription factor SMAD1, which is involved in the regulation of bone morphogenic protein signalling during lung development and pulmonary vascular remodelling [73,74]. Thus, *miR-26a *might be important in controlling essential developmental and physiological events in the lung [75]. Also the *miR-17-92 cluster *is believed to regulate the lung development because its expression is high in embryonic development and steadily declines through development into adulthood [76]. Mice deficient in the *miR-17-92 cluster *died shortly after birth and lung hypoplasia/ventricular septal defects were demonstrated; moreover the absence of the *miR-17-92 cluster *let to upregulation of the pro-apoptotic protein Bim and inhibition of B-cell development [77]. On the other side, the overexpression of the *miR-17-92 cluster *in murine models resulted in an abnormal phenotype manifested by absence of terminal air sacs, which were replaced by highly proliferative, undifferentiated pulmonary epithelium [76]. Other miRNAs found to be involved in the pulmonary homeostasis are members of *let-7 *family [78], *miR-29 *[79], *miR-15 *and *miR-16 *[80,81], which function as tumor suppressors in lung cells. In addition, another miRNA, *miR-223*, has been shown to be crucial for normal granulocyte development and function in the lung [82]. *MiR-223 *mutant mice spontaneously developed neutrophilic lung inflammation with tissue destruction after endotoxin challenge [82].

**Table 4:** MiRNAs involved in physiological processes in the lung.

miRNA	Function (A animal studies, H human studies)	References
*miR-17-92 cluster*	important in lung development and homeostasis (A)	[69,76,77]

*miR-155*	important for normal lung airway remodelling (A)	[70]
	
	alteration of T-cell differentiation (A)	[71]

*miR-26a*	highly expressed within bronchial and alveolar epithelial cells, important for lung development (H)	[75]

*let-7*	highly expressed in normal lung tissue, functions as a tumor suppressor in lung cells (H)	[78]

*miR-29*	functions as tumor suppressor in lung cells (H)	[79]

*miR-15, miR-16*	function as tumor suppressor genes (H)	[80,81]

*miR-223*	control of granulocyte development and function (A)	[82]

*miR-146a/b*	central to the negative feedback regulation of IL-1β-induced inflammation (H)	[83,84]

*miR-200a*, *miR-223*	contribution to the extreme virulence of the r1918 influenza virus (A)	[85]

*miR-17 *family, *miR-574-5p*, *miR-214*	upregulated at the onset of SARS infection (A, H)	[86]

#### Control of pulmonary inflammation and viral infections

Two miRNAs, *miR-146a *and *miR-146b*, have been shown to play central role in the negative feedback regulation of IL-1β-induced inflammation; the mechanism is down-regulation of two proteins IRAK1 and TRAF6 involved in Toll/interleukin-1 receptor (TIR) signalling [83,84]. Also other miRNAs have been shown to regulate the inflammation in mouse lung exposed to aerosolized lipopolysaccharide (LPS): *miR-21*, -*25*, -*27b*, -*100*, -*140*, -*142-3p*, -*181c*, -*187*, -*194*, -*214*, -*223 *and -*224 *[72]. Increase in these miRNAs correlated with the downregulation of pro-inflammatory cytokine production such as TNFα [72]. The deregulation of *miR-155*, the *miR-17-92 cluster *and *miR-223*, miRNAs involved in lung development and homeostasis, resulted in the uncontrolled lung inflammation in murine models [70,77,82].

Based on the studies in murine models, there is evidence that miRNA expression may influence also the course of pulmonary viral infections [85,86]. *MiR-200a *and *miR-223 *were detected in lethal influenza virus infection presumably contributing to the extreme virulence of the r1918 influenza virus [85]. *MiR-17 *family, *miR-574-5p *and *miR-214 *were upregulated at the onset of SARS infection: these miRNAs may help the virus to evade the host immune system and are responsible for effective transmission at the initial stage of viral infection [86].

### Deregulation of miRNAs leads to development of pulmonary diseases

There is evidence that upregulation or downregulation of miRNAs is critical for the lung development/homeostasis and thus may contribute to development of pathological pulmonary conditions, namely to smoking-related diseases including lung cancerogenesis, fibrosis, and other immune-mediated disorders including allergy (Table 5).

**Table 5:** MiRNAs involved in pathological processes in the lung.

miRNA	Function (A animal studies, H human studies)	References
*miR-155, miR-17-92 cluster *	deregulation results in uncontrolled inflammation (A)	[70,71,77]

*miR-21, miR-27b, miR-100, miR-181c, miR-223, miR-224*	increased following exposure to LPS (A)	[72]

*miR-155*	overexpressed in solid tumors, inhibition of tumor suppressor genes (A, H)	[81]

*miR-223*	impaired granulocyte function, regulator of granulocyte production and inflammatory response (A)	[82]

*miR-148a/b, miR-152*	allele-specific regulation of asthma susceptibility HLA-G gene (H)	[87]

*miR-21*	key role in asthma (A)	[88]
	
	overexpressed in solid malignancies (A, H)	[103]
	
	up-regulated in bleomycin-induced fibrosis and IPF (A, H)	[110]

*miR-126*	suppression of the asthmatic phenotype by blockade of *miR-126 *(A)	[89]
	
	downregulated in cystic fibrosis airway epithelial cells (H)	[111]

*let-7, miR-29a, miR-155*	regulation of allergic inflammation in bronchial epithelial cells (A, H)	[90]

*let-7*	pro-inflammatory effect in experimental asthma (A)	[91]
	
	role in lung cancer progression (H)	[99]

*miR-133a*	regulator of expression of RhoA, target for asthma therapy (A, H)	[92,93]

*miR-146a*	reduced expression in COPD fibroblasts (H)	[95]

*miR-218, miR-15a, miR-199b, miR-125a/b, miR-294 *	deregulated due to smoking (A, H)	[96,97]

*miR-218*	tumor suppressor in non-small cell lung cancer (H)	[98]

*miR-17-92 cluster*	overexpressed in lung cancers (H)	[102]

*miR-34*	regulation of apoptosis in lung cancer cells (H)	[105-107]

*miR-210*	overexpressed in lung cancer (H)	[108]

*let-7d*	pro-fibrotic effect in pulmonary fibrosis (A, H)	[109]

#### Immune-mediated lung diseases

Recent studies have implicated the miRNAs in the pathogenesis of immune system mediated pulmonary diseases. Tan and colleagues [87] described that a single nucleotide polymorphism in the 3'UTR of HLA-G, a known asthma-susceptibility gene, disrupts the binding sites of three miRNAs (*miR-148a, miR-148b, miR-152*) targeting this gene. Thus, it is likely that the association of the HLA-G gene to asthma-susceptibility may be due to the allele-specific regulation of this gene by miRNAs [87]. *MiR-21 *is a further miRNA crucially involved in allergic lung inflammation. Its molecular target is IL-12p35, a cytokine contributing to polarization of Th cells toward Th2 cells [88]. *MiR-126 *is another miRNA found to be involved in the pathogenesis of allergic airways disease [89]. The blockade of *miR-126 *suppressed the asthmatic phenotype leading to diminished Th2 responses, suppression of inflammation, reduced airways hyperresponsiveness, inhibition of eosinophil recruitment, and lower mucus hypersecretion [89]. In bronchial epithelial cells stimulated with IL-4 and TNFα, *let-7*, *miR-29a *and *miR-155 *have been involved in the regulation of allergic inflammation [90]. Multiple members of *let-7 *family were also found upregulated in experimental asthma model and the pro-inflammatory role of *let-7 *miRNAs on the allergic cytokine expression was confirmed [91]. Another study showed that expression of RhoA in bronchial smooth muscle cells (BSMCs), a new target for asthma therapy, is negatively regulated by *miR-133a *[92]. The same group later revealed that IL-13 is capable of reducing the *miR-133a *expression in BSMCs and that the *miR-133a *downregulation causes an upregulation of RhoA, presumably resulting in an augmentation of the contraction [93].

#### Smoking-related lung diseases

Lung cancer and chronic obstructive pulmonary disease (COPD) share a common environmental risk factor in cigarette smoke exposure [94]. Although extensive studies of the involvement of miRNAs in lung cancer have been performed, there are only few reports focused on the role of miRNAs in COPD. Recent study on fibroblasts from COPD subjects stimulated *in vitro *with pro-inflammatory cytokines released less *miR-146a *than smokers without COPD [95]. The reduced *miR-146a *expression resulted in prolonged mRNA half-life of cyclooxygenase-2, thus increasing prostaglandin E2 in fibroblasts from COPD subjects [95]. There is evidence that smoking has influence also on other miRNAs. Expression profiling study in the rats exposed to environmental cigarette smoke revealed 24 downregulated miRNAs (especially *let-7 *family, *miR-10, -26, -30, -34, -99, -122, -123, -124, -125, -140, -145, -146, -191, -192, -219, -222, and -223*) when compared to control group [96]. *MiR-294*, a known inhibitor of transcriptional repressor genes, was the only miRNA upregulated in smoke-exposed rats [96]. In another study, bronchial airway epithelial cells from current and never smokers differed in the expression of 28 miRNAs (especially *miR-218, miR-15a, miR-199b, miR-125a/b, miR-294*) in comparison to smokers, whereas the majority of deregulated miRNAs were downregulated in smokers [97]. Similar observation was observed in lung squamous cell carcinoma, where downregulation of *miR-218 *was associated with a history of cigarette smoking [98].

However, the majority of miRNA studies in smoking-related diseases are focused on the role of miRNAs in lung cancer. Altered expression of *miR-155 *and *let-7 *has been reported in lung adenocarcinoma and expression of *let-7 *related to patient survival [99]. Moreover, it has been shown that *let-7 *may also play a role in lung cancer progression [99-101]. Further, increased expression of the *miR-17-92 cluster *has also been detected in lung cancer [102]. Another miRNAs involved in lung cancerogenesis are *miR-21 *and *miR-34 *families. *MiR-21 *was shown to regulate multiple tumor/metastasis suppressor genes in lung solid tumors [103]. *MiR-34a/b/c *have been identified to be a component of the p53 tumor suppressor network: p53 upregulates in response to DNA damage the members of *miR-34 *family [104], thus regulating genes involved in the cell cycle and apoptosis [105-107]. Furthermore, *miR-210 *has been overexpressed in late stages of lung cancer, thus mediated mitochondrial alterations associated with modulation of hypoxia-inducible factor-1 activity [108]. Next, *miR-218 *was identified as a putative tumor suppressor in non-small cell lung cancer [98].

#### Other lung diseases

Recently, it was reported that miRNAs may play pivotal regulatory role also in the fibrotic processes ongoing in the lung: the downregulation of *let-7 d *in idiopathic pulmonary fibrosis (IPF) resulted in the pro-fibrotic effects [109]. Also, upregulation of *miR-21 *was reported in the lungs of IPF patients and in the murine lungs with bleomycin-induced fibrosis, whereas *miR-21 *expression was enhanced by pro-fibrotic TGF-β1 [110]. Another disease associated with miRNA change was cystic fibrosis. Downregulation of *miR-126 *was detected in cystic fibrosis bronchial epithelial cells and its expression correlated with upregulation of TOM1 mRNA both *in vitro *and *in vivo *[111]. TOM1, a *miR-126 *target, was reported to be involved in the regulation of innate immune responses through its involvement in the TLR2/4 and IL-1β and TNF-α-induced signalling pathways [111].

## Conclusion

Small non-coding RNAs (miRNAs) play pivotal role in the posttranscriptional regulation of numerous human genes, mainly via degradation of target mRNAs. There is evidence that the lung has a very specific miRNA expression profile undergoing changes during the lung development. Studies namely in animal models have provided evidence that miRNAs participate in lung homeostasis and play pivotal role also in the control of pulmonary inflammation and viral infections. Recent studies showed evidence that upregulated or downregulated expression of various miRNAs play an active role in the pathogenesis of pulmonary diseases. Specific miRNA expression profiles were characterized for smoking related-diseases including COPD and lung cancer, immune-mediated pulmonary diseases and pulmonary fibrosis. Moreover, several miRNAs crucial for lung development and homeostasis such as *let-7*, *miR-155 *or *miR-19-72 cluster *have been identified to be deregulated in pulmonary allergy, asthma or lung cancer. The knowledge of altered miRNA expression profiles in diseased lung may thus offer new insights in the biology of pulmonary diseases. Moreover, miRNAs may represent attractive novel diagnostic biomarkers mainly due to their higher stability when compared to mRNAs [112] and could potentially provide possibilities for therapeutic intervention [31,113,114].

## Competing interests

The authors declare that they have no competing interests.

## Authors' contributions

All authors wrote and revised the manuscript, and approved the final version.
